# Cardiotocography-Based Experimental Comparison of Artificial Intelligence and Human Judgment in Assessing Fetal Asphyxia During Delivery

**DOI:** 10.7759/cureus.78282

**Published:** 2025-01-31

**Authors:** Kohei Miyata, Chihiro Shibata, Hiroaki Fukunishi, Kazunari Hemmi, Hayato Kinoshita, Toyofumi Hirakawa, Daichi Urushiyama, Masamitsu Kurakazu, Fusanori Yotsumoto

**Affiliations:** 1 Obstetrics and Gynecology, Faculty of Medicine, Fukuoka University, Fukuoka, JPN; 2 Advanced Sciences, Graduate School of Science and Engineering, Hosei University, Tokyo, JPN; 3 Computer Science Program, Graduate School of Bionics, Computer and Media Sciences, Tokyo University of Technology, Tokyo, JPN

**Keywords:** artificial intelligence in medicine, cardiotocography, deep learning artificial intelligence, fetal asphyxia, neonatal acidosis, safe delivery

## Abstract

Cardiotocography (CTG) has long been the standard method for monitoring fetal status during delivery. Despite its widespread use, human error and variability in CTG interpretation contribute to adverse neonatal outcomes, with over 70% of stillbirths, neonatal deaths, and brain injuries potentially avoidable through accurate analysis. Recent advancements in artificial intelligence (AI) offer opportunities to address these challenges by complementing human judgment. This study experimentally compared the diagnostic accuracy of AI and human specialists in predicting fetal asphyxia using CTG data. Machine learning (ML) and deep learning (DL) algorithms were developed and trained on 3,519 CTG datasets. Human specialists independently assessed 50 CTG figures each through web-based questionnaires. A total of 984 CTG figures from singleton pregnancies were evaluated, and outcomes were compared using receiver operating characteristic (ROC) analysis. Human diagnosis achieved the highest area under the curve (AUC) of 0.693 (p = 0.0003), outperforming AI-based methods (ML: AUC = 0.514, p = 0.788; DL: AUC = 0.524, p = 0.662). Although DL-assisted judgment improved sensitivity and identified cases missed by humans, it did not surpass the accuracy of human judgment alone. Combining human and AI predictions yielded a lower AUC (0.693) than human diagnosis alone, but improved specificity (91.92% for humans, 98.03% for humans and DL), highlighting AI's potential to complement human judgment by reducing false-positive rates. Our findings underscore the need for further refinement of AI algorithms and the accumulation of CTG data to enhance diagnostic accuracy. Integrating AI into clinical workflows could reduce human error, optimize resource allocation, and improve neonatal outcomes, particularly in resource-limited settings. These advancements promise a future where AI assists obstetricians in making more objective and accurate decisions during delivery.

## Introduction

Cardiotocography (CTG) has been a traditional and nearly exclusive method for monitoring fetal status during delivery. Multiple guidelines have been established by national societies of obstetrics and gynecology, such as the International Federation of Gynecology and Obstetrics, the American Congress of Obstetricians and Gynecologists, and the Japan Society of Obstetrics and Gynecology, to interpret CTG signals and their implications for maternal and fetal outcomes. Abnormal CTG patterns are usually associated with fetal distress caused by hypoxia or acidosis. An association between low Apgar scores and pathological CTG patterns has been established [1], with more severe pathological CTG linked to neonatal acidosis [2]. Nevertheless, these guidelines exhibit notable disparities [3], and even obstetricians’ opinions vary significantly regarding the classification of CTG [4]. However, CTG monitoring has been used in practice worldwide and analyzed in a number of studies over the past half-century, enabling us to know that the most influential factor for unexpected fetal prognosis has been human error. According to the 2019 ‘Each Baby Counts’ report of the Royal College of Obstetrics and Gynecology, more than 70% of stillbirths, neonatal deaths, and brain injury cases would have had different outcomes with different treatment, and, out of 420 delivery cases, 236 were linked to false interpretation of CTG [5]. Despite half a century of experience in the prediction of fetal prognosis based on CTG, we still could not derive a method to accurately diagnose intrapartum hypoxic-ischemic incidents due to the complex mechanism by which the fetus defends itself. Consequently, there has been a lack of confidence, marked variation in fetal heart rate (FHR) interpretation, defensive practices, unnecessary operative interventions, and a failure to recognize abnormal FHR patterns, resulting in adverse outcomes and expensive litigation [6].

Artificial intelligence (AI) has been widely adopted across various fields, including commerce, distribution, entertainment, marketing, and medicine. Medical AI has been preferred for vision analysis such as for providing assistance with endoscopic or pathological diagnoses. However, AI is expected to be able to diagnose some diseases or implement objective studies in the future, and starting from being an “assistant” of human judgment will be key for acquiring such a role. Early trials and discussions about the priority of diagnostic accuracy have taken place and compared the visually aided analysis with computational analysis [7,8]. In this case, both studies concluded that there is no significant advantage of using one over the other. Moreover, a population-based cohort study found that two-thirds of newborns with low Apgar scores received substandard care due to CTG misinterpretation or delayed response to abnormal patterns [9]. More recently, Petrozziello et al. demonstrated for the first time that deep learning-based visual analysis of non-traditional, feature-labeled CTG datasets is feasible, and argued that the method the authors established improved the prediction of newborn acidemia and complications [6]. Zhao et al. re-plotted the fetal heartbeat dataset based on convolutional neural networks and generated a two-dimensional plot for visual analysis from a traditional linear plot. Their sizable dataset showed 99.05% sensitivity and 97.67% specificity using retrospective 10-fold cross-validation analysis [10]. These studies suggest that certain features of CTG, undetectable to humans, are encoded in the fetal heartbeat, and artificial intelligence was able to find it. Also, such issues gave rise to the notion that the computational diagnostic system should assist obstetricians in making objective and accurate decisions.

In a previous report, we established an automatic assistance device for delivery monitoring to predict asphyxia in newborns. Approximately 4,000 CTG training datasets were used to define abnormal CTG patterns, achieving an area under the curve (AUC) of 0.7. In this study, we experimentally compared the prediction accuracy between AI and specialists in evaluating newborn asphyxia, aiming to assess the potential of AI as a complementary tool to reduce human error.

This article was previously published as a preprint on the Research Square server on July 22, 2022.

## Materials and methods

Patients

CTG data were collected from 38,073 patients immediately prior to delivery at three perinatal medical centers in general hospitals and three obstetrics clinics between 2013 and 2017. Maternal data, including the delivery date, time, and mode, gestational age, and maternal age, as well as neonatal data, including birth weight, gender, pH of the umbilical artery, and Apgar scores at 1 and 5 minutes, were obtained from medical records. All deliveries were singletons. Positive outcomes of newborns were defined as Apgar score <6 at 1 or 5 minutes or umbilical artery pH <7.1.

Data preparation

All CTG datasets were obtained from a Toitu central monitoring system, which generated graphical images of fetal heartbeat at a frequency of 4 Hz and the relative strength of uterine contractions (UC) as comma-separated value (CSV) files. According to the reported method by Zhao et al., regeneration and noise removal from CTG figures were automatically implemented as described previously [10,11]. If any duration of ≥15 s of FHR exclusively contained 0 s, the measurement was discontinued. Any other 0 s measurements were also removed (changed to null). Any FHR of ≥200 beats per minute (bpm) or ≤50 bpm was also removed. If any acceleration and deceleration resulted in an absolute value of >25 bpm within 15 s than the mean bpm of those 15 s, the point was removed as well. Finally, linear interpolation is applied to all removed values and 0 s instances. UC noises were reduced using a moving average window of 60 s. A 30-minute CTG segment up to 20 minutes before delivery was analyzed.

Generation and training of an artificial intelligence judgment system

The generation and training methodology of the AI judgment system used in our study has been explained in detail in our previous report [11]. We established the two AI algorithms used in this study, namely a machine learning algorithm based on the random forest method and a deep learning algorithm based on the CNN model, as discussed. ML was developed as an algorithm that makes judgments based on 44 CTG features, such as baseline variability, acceleration, and deceleration, in a manner similar to human judgment, particularly that of Japanese specialists, and following the guidelines of the Japanese Society of Obstetrics and Gynecology. DL was generated as an algorithm that recognized the processed CTG as one figure and performed the vision analysis. From a total of 38,073 CTG, 3,519 were chosen randomly for the generation of both algorithms because a very small number of positive CTG was obtained. About five times the number of positive CTGs (n=534) were selected among negative CTGs for the subsequent five-fold cross-validation. All codes were written in Python3. Additional information to assist judgment such as gestational weeks was added to improve the prediction ratio.

Comparison between AI and human judgment

**Figure 1:**
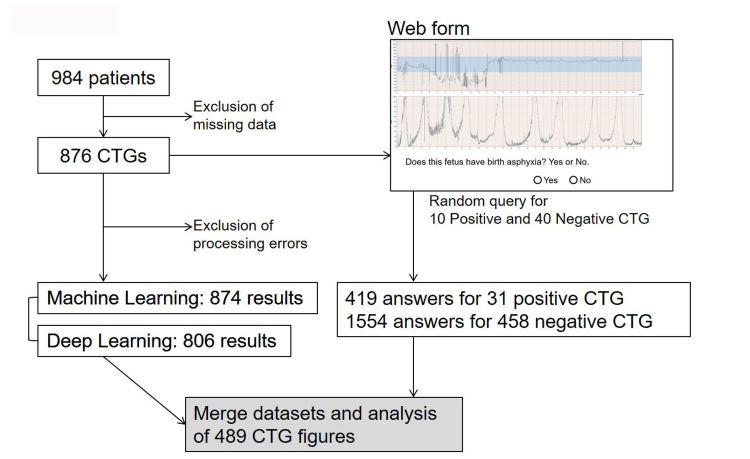
Dataset integration scheme in the present study. CTG: cardiotocography

Another 984 CTGs were obtained from singleton pregnancy deliveries after 34 gestational weeks from the Fukuoka University Hospital from January 2018 to November 2020. All CTG data were processed as described above and the neonatal outcomes were evaluated as positive or negative. Since the results of the DL algorithm were obtained as a prediction ratio, a 0.50 and higher score was recognized as a positive judgment. To collect the human judgment data, we created a query form on the web server of the Tokyo University of Technology and asked 34 obstetricians and 22 midwives to evaluate 50 CTG figures each and indicate whether the newborn was predicted to be suffering from asphyxia. The prediction ratio of CTG figures was categorized as positive which could predict a neonatal asphyxia state according to a newborn Apgar score of <6 either at 1 or at 5 min and a pH of <7.1 of umbilical artery gas analysis, whereas the remaining ones were deemed negative. Each category was displayed randomly, and its distribution and number were described in Figure 1. Sensitivity, specificity, accuracy, negative predictive value, and precision were calculated for human, machine learning, deep learning, and human-deep learning collaborative analysis, respectively. The percentage of human correct responses for each CTG was calculated separately for physicians and midwives. For the combined prediction of DL and human judgment, the predictive value was determined as the product of the DL's predictive value and the percentage of correct responses from the human for each CTG. Receiver operating characteristic analyses were used for statistical tests.

## Results

The experimental design is illustrated in Figure 1. We utilized 876 CTG figures from 984 patients and collected 1,973 human assessments for 489 CTG cases using web-based questionnaires completed by specialists. Each specialist assessed 50 randomly selected CTG figures, comprising 10 positive and 40 negative cases. The results from the human questionnaires were calculated based on the prediction ratio for each CTG figure. Additionally, 874 of the 876 processed CTG figures were analyzed using both ML and DL algorithms. The ML algorithm results were scored as "1" for positive and "0" for negative predictions, while DL calculated prediction ratios for newborn asphyxia based on CTG figures. Results from both human and AI analyses were collected for 489 figures and integrated with their corresponding clinical features for sequential analysis. The characteristics of the patients are shown in Table 1. This table presents the characteristics of patients included in the study, classified based on whether they had negative or positive outcomes regarding newborn asphyxia. Younger maternal age (<35 years), fewer pregnancies (Gravida 0), and full-term gestational age (37-40 weeks) were more common among patients with negative outcomes. Vaginal delivery was the predominant mode, followed by cesarean section. Positive outcomes were less frequent across all categories, with the majority of cases concentrated in the ≥35 years age group, nulliparous patients, and those delivering at term.

**Table 1:** The background information of the patients enrolled in this study. CS: cesarean section

Characteristics	Categories	Negative	Positive
Maternal age (years)	<35	156	14
	≥35	302	17
Gravida	0	259	17
	1	135	6
	2	47	5
	>3	17	3
Gestational weeks	34-36	83	7
	37-40	348	23
	41-42	27	1
Delivery mode	Vaginal	301	12
	Vacuum	60	6
	Forceps	7	0
	CS	90	13

**Figure 2:**
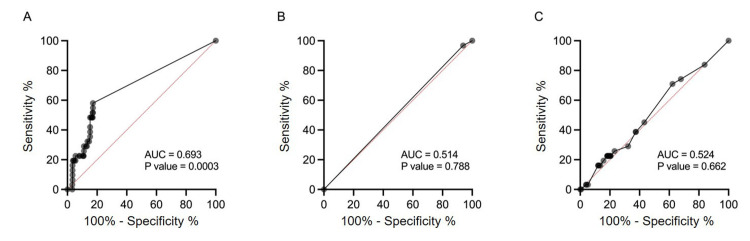
ROC analysis of estimated fetal asphyxia ratio with each method alone. ROC curves of estimated newborn asphyxia ratios calculated for humans (A), deep learning (B), and machine learning (C) diagnoses are presented. ROC: receiver operating characteristic; AUC: area under the curve

The performance metrics for each analysis method are shown in Table 2, and the diagnostic outcomes for asphyxia by each judgment method are presented in Table 3. Human- and DL-mediated diagnoses showed higher sensitivity (22.58%) compared with ML-mediated ones (3.23%). Human analysis exhibited high performance for all calculated parameters, though specificity and accuracy were higher in ML analyses (93.89% and 88.14%, respectively). ROC analysis was performed to evaluate the diagnostic accuracy of both human and AI assessments. The highest AUC (0.6931) and p-value (0.0003) were observed in human diagnoses (Figure 2A), whereas the existent AI judgment system did not diagnose newborn asphyxia reliably (ML AUC of 0.5144, p = 0.7877; DL AUC of 0.5235, p = 0.6619) (Figures 2B, 2C). Although human results showed a higher AUC, the highest likelihood ratio observed for AI was 5.214, indicating that AI alone is not yet suitable for diagnosis [12]. These results indicated that unassisted human judgment was the most useful means of estimating newborn asphyxia. DL frequently misclassified negative CTG as positive, while ML often misclassified positive CTG as negative.

**Table 2:** Performance comparison of the proposed methods. DL: deep learning; ML: machine learning

Analysis	Sensitivity (%)	Specificity (%)	Precision (%)	Negative predictive value (%)	Accuracy (%)
Human	22.58	91.92	15.91	94.61	87.53
ML	3.23	93.89	3.45	93.48	88.14
DL	22.58	81.88	7.78	93.98	78.12
Human + DL	12.90	94.76	14.29	94.14	89.57
Human x DL	9.68	98.03	25.00	94.13	92.43

**Table 3:** Distribution of the fetal status for each method and prediction result. DL: deep learning; ML: machine learning

Analysis	Prediction	No asphyxia	Asphyxia
Human only	Negative	421	24
	Positive	37	7
ML only	Negative	430	30
	Positive	28	1
DL only	Negative	375	24
	Positive	83	7
Human and DL combination	Negative	449	28
	Positive	9	3

**Figure 3:**
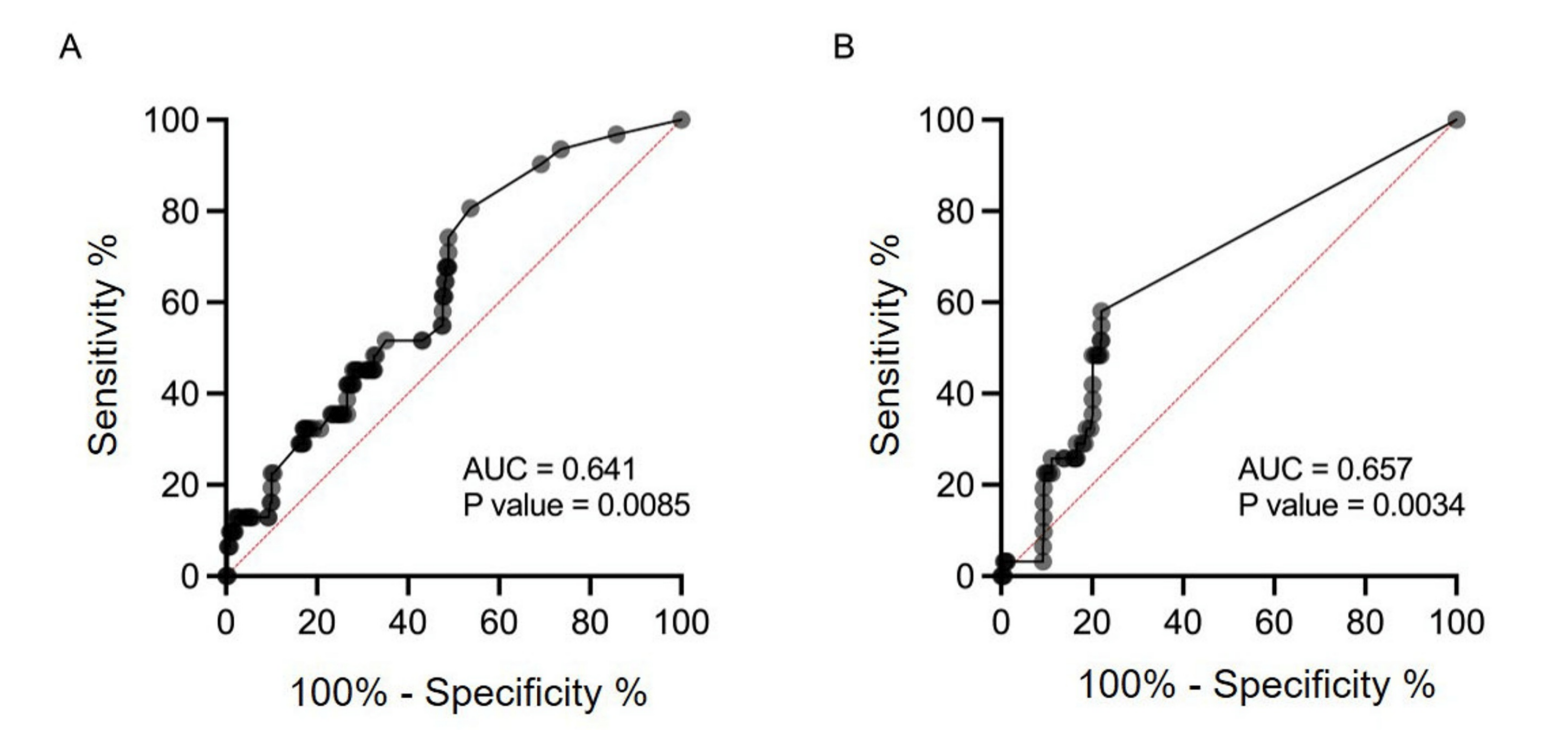
ROC analysis of the aggregated estimated ratio calculated for humans and for each of the AI analyses. The ROC curves are presented based on the estimated newborn asphyxia ratio calculated for human- and deep learning-mediated diagnosis (A) or human- and machine learning-mediated diagnosis (B). ROC: receiver operating characteristic; AUC: area under the curve

To assess the effect of human input into AI, the human and AI estimated ratio was added to each other and analyzed. The aggregated prediction ratio from human and DL judgments showed a lower AUC (0.6411) and higher p-value (p=0.0085) compared to human judgment alone. However, a higher likelihood ratio (0-14.77) was achieved, altering the AUC curve (Figure 3A). The combined prediction ratio from human and ML analyses performed worse than human judgment alone in terms of accuracy (AUC=0.657) and produced a lower likelihood ratio (0-4.925) compared to human results alone (Figure 3B). These results suggested that the DL method based on visual analysis could complement human judgment in screening for positive CTG figures that have been misdiagnosed by humans.

## Discussion

This study demonstrated that AI can complement human judgment in predicting newborn asphyxia from CTG data during delivery. Human judgment exhibited the highest AUC; however, AI assistance improved the specificity of newborn asphyxia predictions.

Several studies in this field have addressed specific issues with CTG, including human error and the lack of objectivity. Researchers and clinicians have expected to resolve this problem using computational analysis. Computational analysis of CTG traces, with the purpose of alerting clinicians, has been shown to be beneficial in predicting the pH of the umbilical artery at birth, even when it simply alerts for the presence of normal features [13]. With advancements in computational analysis technologies, AI is tasked with developing novel automated algorithms.

AI-based CTG analysis has demonstrated promising levels of accuracy in various studies. For instance, Zhao et al. generated two-dimensional fetal heart rate plots based on convolutional neural networks (CNN), rather than traditional one-dimensional CTG plots, and achieved an AUC of 0.9836 using 9,164 CTG obtained ≤90 min before delivery [10]. Petrozziello et al. established a newborn asphyxia detection system based on CNN analysis by generating a two-dimensional plot from 35,429 CTG figures which achieved an AUC of 0.92 [6]. This big-data analysis showed an accuracy as high as that of Zhao et al., using five-fold cross-validation [10]. According to our previous work, the AI evaluation system used in this study exhibited high performance with an AUC of 0.949 using a five-fold cross-validation analysis. However, although high-performance algorithms were established with the 3,519 CTG figures used, the prediction accuracy has been insufficient in our study. The difference in prediction accuracy between the five-fold cross-validation analysis and the new cohort used in this study remains unexplained. The cohort size is a strongly influencing factor for validation analysis since the CTGs were very often diagnosed as positive. Further accumulation of CTG was necessary for the establishment of an AI judgment system and its accurate validation. Traditional human judgment methods for CTG, such as delivery monitoring, have been reported to exhibit false-positive rates as high as 60% and significant intraobserver variability [10].

Overinterpretation of CTG figures is common and the direct cause of implementing unnecessary cesarean sections [6]. On the contrary, misinterpretation of intrapartum fetal monitoring may lead to hypoxia-induced encephalopathy, which has an overall incidence rate of 0.37 per 1,000 live births in Japan [14]. There were many more incidences of this adverse event in other countries, for example, 5.14 per 1,000 live births in the United Kingdom and 1.1 per 1,000 live births in Sweden [15,16]. The neonatal mortality rate is significantly higher in developing countries compared to developed ones, with a correspondingly higher incidence of fetal hypoxia, which has been estimated as higher in these countries [17]. Inadequate medical systems which included poor human resources, education, medical devices, and facilities, have been the cause of poor neonatal prognosis. The development of CTG monitoring during delivery using automated devices, which are able to ameliorate the shortage of human resources or prevent misinterpretation, is required in both situations to promote neonatal welfare.

The accuracy of the diagnosis method based on AI in this study has been definitely insufficient, especially for ML. The ML method employed traditional criteria for CTG evaluation, including variability, accelerations, and decelerations, similar to human judgment. The judgment of humans and ML presented much higher accuracy - seven out of 31 truly positive CTG were detected by means of human judgment and only one out of 31 truly positive CTG was detected by ML alone. DL detected seven out of 31 true-positive CTG cases in combination with human judgment; however, this combination did not improve the overall AUC in the ROC analysis. The aggregation of prediction ratio obtained from human and DL judgments definitely promoted sensitivity, and even the total accuracy was found to be lower than human judgment alone. Therefore, DL will be able to achieve a decrease in the high false-positive ratio of human judgment. Interestingly, four of the seven CTG cases were identified by both humans and DL, while the remaining three positive cases identified by DL were missed by humans. This suggested that there were some unknown features that only AI could detect to predict positive CTG.

A recently published approach utilized wavelet transform for CTG analysis, offering an effective method for multi-resolution analysis with both time and frequency orientations using advanced signal processing [18]. Instead of using traditional linear analysis of CTG figures and their features such as variability, acceleration, or deceleration, the merit of the wavelet transform lies in that it is a better way to observe and capture the local hidden characteristic information of the fetal heart rate signal as a function of both time and frequency [19]. Though humans have traditionally been diagnosed using linear CTG figures with uterine contractions, processing and assessment processes should be integrated to simplify the analysis for AI.

Limitations

In this study, the relatively low frequency of fetal distress resulted in a disproportionately small number of positive cases compared to negative cases, which may have limited the accuracy of the artificial intelligence analysis. Although more than 30,000 CTG diagrams were collected, an even larger dataset is necessary to further enhance the accuracy of AI learning.

## Conclusions

In conclusion, we demonstrated that while the human judgment of cardiotocography (CTG) exhibited the highest diagnostic accuracy for predicting newborn asphyxia, artificial intelligence (AI) systems, particularly deep learning (DL), showed the potential to complement human interpretation by identifying cases missed by specialists. However, the current AI methods are insufficiently accurate to replace human judgment, particularly for machine learning (ML). Combining human and AI analyses improved specificity but did not significantly enhance overall diagnostic accuracy. These findings suggest that AI can assist in reducing human errors and false-positive rates, potentially leading to safer delivery outcomes. Further refinement of AI algorithms, along with larger datasets, is essential to improve their reliability and establish their role as a valuable tool in perinatal care.
